# Genetic deletion of Krüppel-like factor 11 aggravates traumatic brain injury

**DOI:** 10.1186/s12974-022-02638-0

**Published:** 2022-11-19

**Authors:** Chao Zhou, Ping Sun, Milton H. Hamblin, Ke-Jie Yin

**Affiliations:** 1 Geriatric Research, Education and Clinical Center, Veterans Affairs Pittsburgh Healthcare System, Pittsburgh, PA 15261 USA; 2 Department of Neurology, School of Medicine, Pittsburgh Institute of Brain Disorders & Recovery, University of Pittsburgh, S514 BST, 200 Lothrop Street, Pittsburgh, PA 15213 USA; 3 Tulane University Health Sciences Center, Tulane University, New Orleans, LA 70112 USA; 4 College of Pharmacy, Xavier University of Louisiana, New Orleans, LA 70125 USA

**Keywords:** Krüppel-like factor 11, Neurobehavioral deficits, White matter injury, Grey matter injury, Neuroinflammation, Cytokines

## Abstract

**Background:**

The long-term functional recovery of traumatic brain injury (TBI) is hampered by pathological events, such as parenchymal neuroinflammation, neuronal death, and white matter injury. Krüppel-like transcription factor 11 (KLF 11) belongs to the zinc finger family of transcription factors and actively participates in various pathophysiological processes in neurological disorders. Up to now, the role and molecular mechanisms of KLF11 in regulating the pathogenesis of brain trauma is poorly understood.

**Methods:**

KLF11 knockout (KO) and wild-type (WT) mice were subjected to experimental TBI, and sensorimotor and cognitive functions were evaluated by rotarod, adhesive tape removal, foot fault, water maze, and passive avoidance tests. Brain tissue loss/neuronal death was examined by MAP2 and NeuN immunostaining, and Cresyl violet staining. White matter injury was assessed by Luxol fast blue staining, and also MBP/SMI32 and Caspr/Nav1.6 immunostaining. Activation of cerebral glial cells and infiltration of blood-borne immune cells were detected by GFAP, Iba-1/CD16/32, Iba-1/CD206, Ly-6B, and F4/80 immunostaining. Brian parenchymal inflammatory cytokines were measured with inflammatory array kits.

**Results:**

Genetic deletion of KLF11 worsened brain trauma-induced sensorimotor and cognitive deficits, brain tissue loss and neuronal death, and white matter injury in mice. KLF11 genetic deficiency in mice also accelerated post-trauma astrocytic activation, promoted microglial polarization to a pro-inflammatory phenotype, and increased the infiltration of peripheral neutrophils and macrophages into the brain parenchyma. Mechanistically, loss-of-KLF11 function was found to directly increase the expression of pro-inflammatory cytokines in the brains of TBI mice.

**Conclusion:**

KLF11 acts as a novel protective factor in TBI. KLF11 genetic deficiency in mice aggravated the neuroinflammatory responses, grey and white matter injury, and impaired long-term sensorimotor and cognitive recovery. Elucidating the functional importance of KLF11 in TBI may lead us to discover novel pharmacological targets for the development of effective therapies against brain trauma.

**Supplementary Information:**

The online version contains supplementary material available at 10.1186/s12974-022-02638-0.

## Background

Traumatic brain injury (TBI) is an incurable neurological disease with a high rate of morbidity and mortality in young adults worldwide [1, 2]. Patients who survive TBI suffer from various neurofunctional deficits, including but not limited to headaches, slurred speech, aphasia, sensory and motor deficits, cognitive (learning and memory) impairment, and emotional (irritability and aggression) dysfunction [1, 3]. Brain trauma is characterized by neuronal death, diffuse axonal damage, and demyelination [4, 5]. Pathologically, mechanical injury induces disruption of the blood–brain barrier (BBB) [6] along with a reduction of regional cerebral blood flow (CBF) in TBI brains [7, 8], which subsequently causes infiltration of peripheral blood immune cells such as neutrophils and macrophages to the brain parenchyma [9, 10]. TBI also elicits proliferation, activation, and migration of astrocytes and microglia in pericontusional brain regions [11]. It is well known that microglial cells are able to be polarized to both pro-inflammatory (M1) and inflammatory-resolving (M2) phenotypes after TBI [12, 13]. Activation of resident microglia, astrocytes, and infiltrated immune cells triggers the release of abundant inflammatory cytokines in post-trauma brains [11, 14]. These pathological cascades synergistically account for the sensorimotor deficits and cognitive impairment after TBI [3, 15]. Nowadays, long-lasting neuroprotection after TBI is still an unmet need and effective therapeutic strategies for TBI are urgently required.

Krüppel-like factors (KLFs) belong to the zinc finger family of transcription factors. Previous studies demonstrated that different KLF members (KLF1–18) regulate numerous cell functions including proliferation, differentiation, migration, metabolism, apoptosis, and cell death by transcriptional activation or suppression of target genes [16]. Recently, Li et al. demonstrated that KLF7 protects hippocampal neurons against TBI through activation of the JAK2/STAT3 signaling pathway [17]. We have previously demonstrated that KLF11 was significantly downregulated in ischemic stroke and genetic deletion of KLF11 aggravated ischemic brain damage and worsened neurobehavioral performance in mice [18]. In contrast, endothelium-targeted transgenic overexpression of KLF11 preserved BBB structural and functional integrity, and therefore, conferred brain protection in ischemic stroke [19]. Additionally, we also elucidated that KLF11 is required for peroxisome proliferator-activated receptor gamma (PPARγ) to transcriptionally suppress microRNA-15a/16-1 and subsequently protect against ischemic stimuli [20]. However, the molecular events and regulatory roles of KLF11 in brain trauma remain unclear.

In the present study, we sought to uncover the essential role of KLF11 in the regulation of cerebral glial cell activation, inflammatory responses, grey matter and white matter injury, and long-term sensorimotor and cognitive dysfunctions after TBI.

## Materials and methods

### Animals and experimental design

All procedures using laboratory animals were approved by both the Department of Veterans Affairs Pittsburgh Healthcare System (VAPHS) and the University of Pittsburgh Institutional Animal Care and Use Committees (IACUCs) and conducted consistently with the National Institutes of Health Guide for the Care and Use of Laboratory Animals. Mice were housed in groups of four per cage in a temperature- and humidity-controlled animal facility with a 12-h light–dark cycle. Food and water were available ad libitum. All efforts were made to minimize animal suffering and the number of animals euthanized.

KLF11 knockout (KO) mice were kindly provided by Dr. Eugene Chen [20]. Both KLF11 KO and wild-type (WT) mice were randomly assigned to either the sham or traumatic brain injury group by a lottery box. All outcomes were performed by independent investigators blinded to procedures and mouse types.

### Murine model of traumatic brain injury

Experimental traumatic brain injury (TBI) was induced in KLF11 KO and littermate WT mice (male, 8–12w, 23–30 g) by a controlled cortical impact (CCI) device (Precision Systems and Instrumentation, Fairfax, VA, USA) as described previously [21, 22]. Briefly, mice were continuously anesthetized with 1.5–3% isoflurane in a mixture of 25% O_2_ and 74% N_2_O during surgical procedures. The mouse head was firmly mounted on the CCI apparatus in the prone position. A midline scalp incision was made to expose the skull. Then, craniotomy was conducted over the right parietotemporal cortex (diameter of 4.0 mm; centered 0.5 mm anterior and 2.0 mm lateral to Bregma) using a motorized drill. CCI was performed using the CCI device with a 3-mm (diameter) flat-tipped impactor to compress the exposed dura mater and underlying brain to a depth of 1.5 mm at a peak velocity of 3.5–3.7 m/s for a dwell time of 150 ms. After CCI, the skull gap was sealed with Koldmount cement (Vernon Benshoff, Albany, NY, USA), and the scalp incision was sealed with a 6-0 suture. Rectal temperature was maintained at 37.0 ± 0.5 °C and heart rate was monitored during surgery. The animal numbers and mortality rates of this study are listed in Additional file 2: Table S1.

### Assessments of sensorimotor and cognitive functions

A panel of neurobehavioral tests was performed to assess sensorimotor and cognitive functions after TBI. On each testing day, we conducted different neurobehavioral tests in the same sequence and at the same time each day to avoid the effects of the biological clock and light cycle on neurobehavioral results. The sequence of sensorimotor behavioral tests is as follows: adhesive tape removal test, foot fault test, and rotarod test.

Rotarod test: Long-term sensorimotor function was evaluated by the rotarod test before and 3, 5, 7, 14, 21, and 28 days after TBI as described previously [23]. Briefly, experimental mice were placed into a rotating drum (IITC Life Science Inc.) accelerating from 5 to 40 rpm within 5 min. Each mouse was examined 3–4 times/day for 3 consecutive days before and up to 28 d after operation with a 5-min interval between each trial. The mean time of 3–4 trials each day on the rod (latency to fall) before surgery (baseline) and at selected time points after operation were recorded.

Adhesive tape removal test: Forepaw sensorimotor function was evaluated by the adhesive tape removal test as described previously [23]. Briefly, experimental mice were placed into a transparent Plexiglas cylinder (30 cm tall by 20 cm diameter) for a 60-s habituation period before testing. Then, a piece of adhesive tape (0.3 × 0.4 cm) was placed on the left hairless part of the forepaws. The time when the adhesive tape was touched (time to touch) and completely removed (time to remove) from the forepaw was recorded. Three trials per day were conducted for 3 consecutive days before surgery and at selected time points after surgery. The maximum time between touch and removal was recorded as 120 s if the mouse did not touch or remove the adhesive tape.

Foot fault test: Forepaw and hindpaw sensorimotor functions were examined by the foot fault test as described previously [24]. Mice were allowed to walk freely on a metal grid surface for 3 min, and a foot fault was counted when the forepaw or hindpaw fell or slipped between the wires. One trial per day was conducted 1 day before surgery and at selected time points after operation. Data were expressed as the percentage of error steps to the total moving steps of the contralateral forepaw.

Morris water maze (MWM): Long-term spatial learning and reference memory were examined by the MWM test at 22–27 days after TBI as described previously [23, 25]. Before surgery, mice were placed into a water tank to explore freely for 90 s. Mice that were motionless while exploring the water tank were excluded from formal testing. In the place navigation (22–26 d, learning) phase, a circular platform was immersed 1.5–2 mm below the water surface and placed in the middle of the 4th quadrant (target quadrant). Mice were released to the pool from the 1st, 2nd, and 3rd quadrants in a random order each day and the times to find the platform (latency to platform) in each trial were recorded. The probe test (27 d, memory) on the 6th day was conducted by removing the hidden platform. Mice were allowed to explore for 60 s in the tank and the swimming speed and time expended in the target quadrant were recorded. ANYMAZE software and a video tracking system were used for recording the MWM test.

Passive avoidance: Long-term cognitive function was also examined by the passive avoidance test as described previously [21, 26]. Briefly, in the training phase (29 d), mice were placed into the lightbox and acclimatized for 60 s. Then, the door was raised between the light and dark box. After mice crossed over the door to the dark side, the door was dropped, and the mice received a footshock (0.4–1.6 mA). Mice were allowed to stay in the dark box for 30 s to strengthen the connections between the aversive stimulus and dark environment. On testing day (30 d), mice were placed into the lightbox and the door was raised again. Three trials on testing day were conducted and the mean time to enter the dark box within 180 s in three trials was recorded.

### Histological staining

Experimental mice were deeply anesthetized and transcardially perfused with 30 ml of saline followed with 30 ml 4% paraformaldehyde. Next, mouse brains were dissected and post-fixed in 4% paraformaldehyde overnight at 4 °C and immersed in 30% sucrose in 0.1 M phosphate buffer for another 2 days. After completely dropping to the bottom of 30% sucrose solution, mouse brains were cut into 25-µm coronal sections by using a microtome (ThermoFisher HM450) and brain sections were preserved in cryoprotectant at − 20 °C until further use.

Cresyl violet (CV) staining: CV histological staining was performed to examine TBI-induced neuronal death [23]. Briefly, brain sections were deionized and immersed in Cresyl violet solution followed by distilled water, 70% alcohol, 95% alcohol, 100% alcohol, and xylene. Three randomly selected regions of interest (0.2 mm × 0.2 mm) from 3 consecutive sections of the pericontusional cerebral cortex and hippocampus were used for examining the areas of CV-stained neurons in each brain. Images were taken with an EVOS M7000 microscope, and the percentage of CV-positive stained areas were calculated and analyzed by using Image J Software.

Luxol fast blue (LFB) staining: LFB histological staining was conducted to evaluate white matter injury after TBI [23]. Briefly, brain sections were immersed in LFB solution, decolored with 70% alcohol and 0.05% Li_2_CO_3_, and dehydrated with graded ethanol. Images were taken by using an EVOS M7000 microscope. Three randomly selected microscopic fields from 3 consecutive sections were used for calculating the relative OD value in the pericontusional cerebral cortex, external capsule, and striatum regions of each brain with Image J software.

### Immunofluorescence staining

Immunofluorescence staining was performed as previously described [27]. Briefly, brain sections were washed 3 times for 5 min with PBS in a 24-well plate, permeabilized once for 20 min with 1% PBST (1% Triton-X 100 in PBS) and washed two times for 5 min with 0.3% PBST. The free-floating sections were then blocked with 5% normal donkey serum in 0.3% PBST for 1 h at room temperature. Then, mouse brain sections were incubated with primary antibodies (diluted in 0.3% PBST) followed by secondary antibodies accordingly. Primary antibodies used in this study, corresponding dilution factors, and vendor information are listed in Additional file 3: Table S2. Images were captured by a confocal microscope (A1R, Nikon, Japan). The mean fluorescence intensities of MBP/SMI32, immunostained area of NeuN or GFAP, and immunopositive cell numbers of Iba-1/CD16/32, Iba-1/CD206, F4/80, or Ly-6B were processed for analyses with Image J software. Three randomly selected microscopic fields from 3 consecutive sections in the pericontusional cortex, cerebral cortex, external capsule, striatum, and hippocampus were analyzed for each brain. MAP2 immunostaining of 6 coronal brain sections (bregma: + 0.98 mm to − 1.58 mm) was visualized by an EVOS M7000 microscope and used for the assessment of TBI-induced brain atrophy. Brain atrophy (%) = 100 (contralateral hemisphere volume − ipsilateral hemisphere volume)/contralateral hemisphere volume. The nodes of Ranvier (NOR) were examined by co-immunostaining Caspr with Nav1.6. The number of nodes, paranode length, and length of the paranodal gap in the external capsule was counted from 15 to 20 NORs in each brain [28].

### Protein extraction and inflammatory antibody array

Total protein was extracted from the impacted mouse brain hemisphere at 3 d after TBI or sham operation as described previously [28]. A total of 250 µg proteins per sample were used to examine the expression of 40 inflammatory mediators using a RayBiotech mouse inflammatory antibody array kit (AAM-INF-1-8, GA, USA). The relative expression levels of the 40 inflammatory mediators were normalized to the positive controls in each membrane.

### Statistical analyses

The normality of all data in this study was evaluated with the Shapiro–Wilk test. All data in this study are expressed as mean ± SD with dots and analyzed with GraphPad Prism 9 (GraphPad Software, CA, USA). For data that meet the Shapiro–Wilk normality test and Brown–Forsythe homogeneity of variance test of multiple-group comparisons, one-way or two-way ANOVA was used followed by Tukey’s post hoc test. Welch ANOVA test and Dunnett T3 post hoc tests were used when the variances were heterogeneous. The Kruskal–Wallis test was used when data distribution did not meet normal Gaussian distribution. A two-tailed *t*-test was used for a two-group comparison. Two-tailed Pearson correlation analyses were utilized for correlation analysis in R (Version 4.1.1). A *p* ≤ 0.05 was considered statistically significant.

## Results

### Genetic deletion of KLF11 exacerbates long-term sensorimotor deficits after TBI in mice

**Fig. 1:**
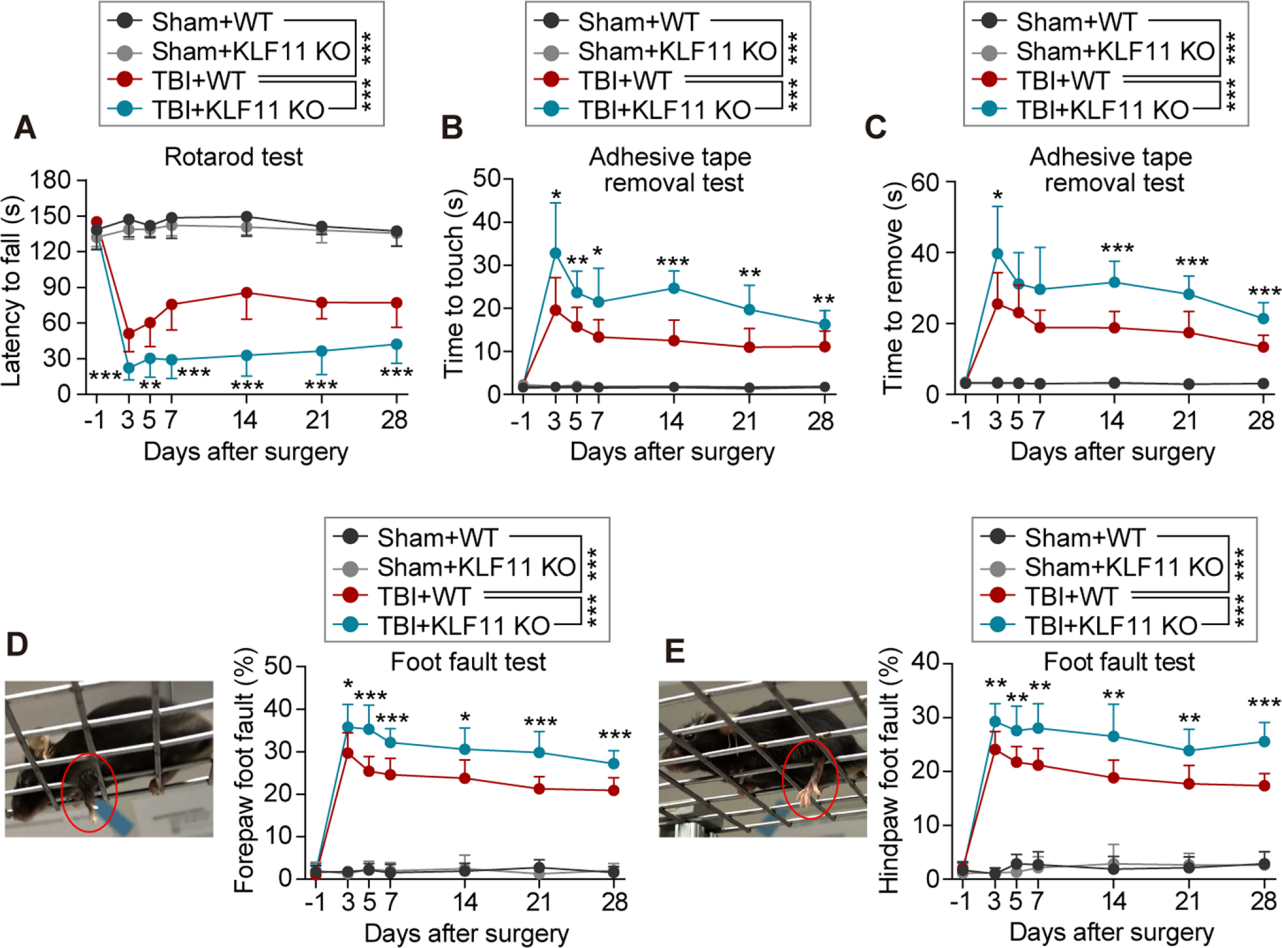
Genetic deletion of KLF11 aggravates long-term sensorimotor deficits in mice after TBI. Experimental TBI was induced in KLF11 KO and WT mice by unilateral controlled cortical impact, followed by a 30-d survival period. Long-term sensorimotor function was examined by the rotarod test, adhesive tape removal test, and foot fault test at the indicated time points (− 1, 3, 5, 7, 14, 21, and 28 days after operation). **A** The latency to fall in the rotarod test. **B** The time to touch and **C** time to remove the tape in the adhesive tape removal test. **D** The forepaw foot fault rate and **E** hindpaw foot fault rate in the foot fault test. Data are presented as mean ± SD, *n* = 11–12/group. Statistical analyses were performed by two-way ANOVA with Tukey’s post hoc test. *p < 0.05, **p < 0.01, and ***p < 0.001 versus TBI + WT group

The overall experimental design of this study is shown in Additional file 1: Fig. S1. To explore the effect of KLF11 genetic deletion on sensorimotor function in mice after brain trauma, either CCI or sham operations were conducted in KLF11 KO and WT mice. Experimental mice were subjected to a panel of sensorimotor behavioral tests including rotarod, adhesive tape removal, and foot fault tests before and 3, 5, 7, 14, 21, and 28 d after operation. No significant difference was determined in sensorimotor function between KLF11 KO and WT mice under sham conditions. WT mice with CCI exhibited a reduced time to fall in the rotarod test (Fig. 1A), increased time to touch (Fig. 1B) and remove (Fig. 1C) in the adhesive tape removal test, and an increased forepaw (Fig. 1D) and hindpaw foot fault rate (Fig. 1E) in the foot fault test at 3–28 d after TBI in comparison with sham controls, indicating that severe sensorimotor deficits were induced in mice by TBI. Genetic deletion of KLF11 further exacerbated sensorimotor deficits, showing less time to stay on the rotarod (Fig. 1A), longer time to touch (Fig. 1B) and remove (Fig. 1C) in the adhesive tape removal test, and more faulted forepaw or hindpaw steps (Fig. 1D, E) in the foot fault test compared with WT controls 3–28 d after TBI. These data demonstrated that genetic deletion of KLF11 aggravates short-term and long-term sensorimotor deficits in mice after TBI.

### Genetic deletion of KLF11 aggravates long-term cognitive impairment in mice after TBI

**Fig. 2:**
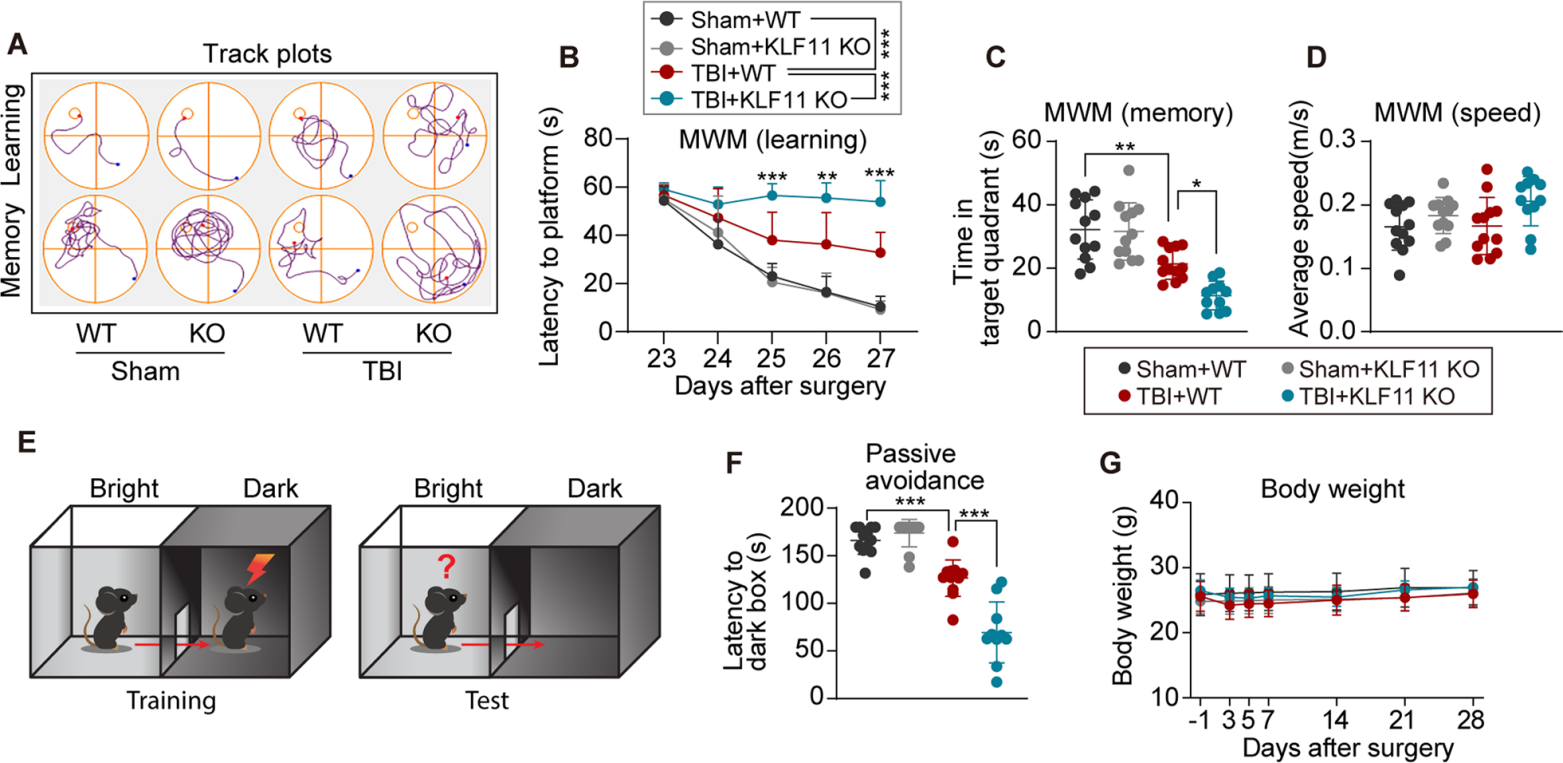
Genetic deletion of KLF11 exacerbates long-term cognitive impairment in mice after TBI. KLF11 KO and WT mice were subjected to TBI or sham operation, followed by a 30 d survival period. Long-term cognitive function was evaluated by the Morris water maze test at 23–28 d after TBI and the passive avoidance test at 29–30 d after TBI. **A** Swim paths of learning and memory phases during the water maze test. **B** The latency to find the hidden platform in the place navigation phase (learning). **C** The swim time in the target quadrant in the probe test (memory). **D** Average swimming speed in the water maze test. **E** Graphic protocol of the passive avoidance test. **F** The latency of entering into the dark box in the passive avoidance test. **G** Body weight changes 28 d after TBI or sham operation. Data are presented as mean ± SD, *n* = 11–12/group. Statistical analyses were performed by two-way ANOVA with Tukey post hoc test (**B**, **G**) and one-way ANOVA with Tukey post hoc test (**C**, **D**, **F**). *p < 0.05, **p < 0.01, and ***p < 0.001 versus TBI + WT group

We further examined whether KLF11 genetic deletion affects TBI-induced cognitive impairment in mice. The Morris water maze (MWM) test and passive avoidance test were conducted to assess learning and memory functions in experimental mice 22–30 d after TBI. No significant difference in cognitive function was observed between KLF11 KO and WT mice under sham conditions, as evidenced by showing similar performing patterns in MWM and passive avoidance tasks. TBI mice exhibited longer time to find the hidden platform in the learning phase compared with sham controls (Fig. 2A, B). After TBI, KLF11 KO mice spent much more time to find the platform in comparison with WT controls, suggesting a declined learning ability in mice with genetic deletion of KLF11 (Fig. 2A, B). In the probe test, TBI-induced WT mice spent shorter time in the target quadrant compared with sham controls (Fig. 2A, C), indicating impaired post-trauma reference memory. Genetic deletion of KLF11 further exacerbated memory impairment in mice after TBI when compared with WT controls, as evidenced by showing less exploration time in the target quadrant. There were no significant differences on average swimming speed among all experimental groups, which excluded the effect of motor function on MWM outcomes (Fig. 2D). In the passive avoidance test (Fig. 2E), after aversive footshock stimuli in the training phase (29 d after TBI), sham-operated mice seldom crossed over the door to the dark environment because of the painful memory. However, TBI mice presented a reduced waiting time before entering the dark box compared with sham controls (Fig. 2F). Further, KLF11 KO mice exhibited an even shorter time staying in the lightbox regardless of footshock stimulus when compared with WT controls, suggesting a much greater decline in memory ability (Fig. 2F). Additionally, there was no significant difference in body weight between KLF11 KO and WT mice under TBI or sham conditions (Fig. 2G). These data suggest that genetic deletion of KLF11 accelerates cognitive impairment in mice after TBI.

### Genetic deletion of KLF11 exacerbates brain atrophy and neuronal death in mice after TBI

**Fig. 3:**
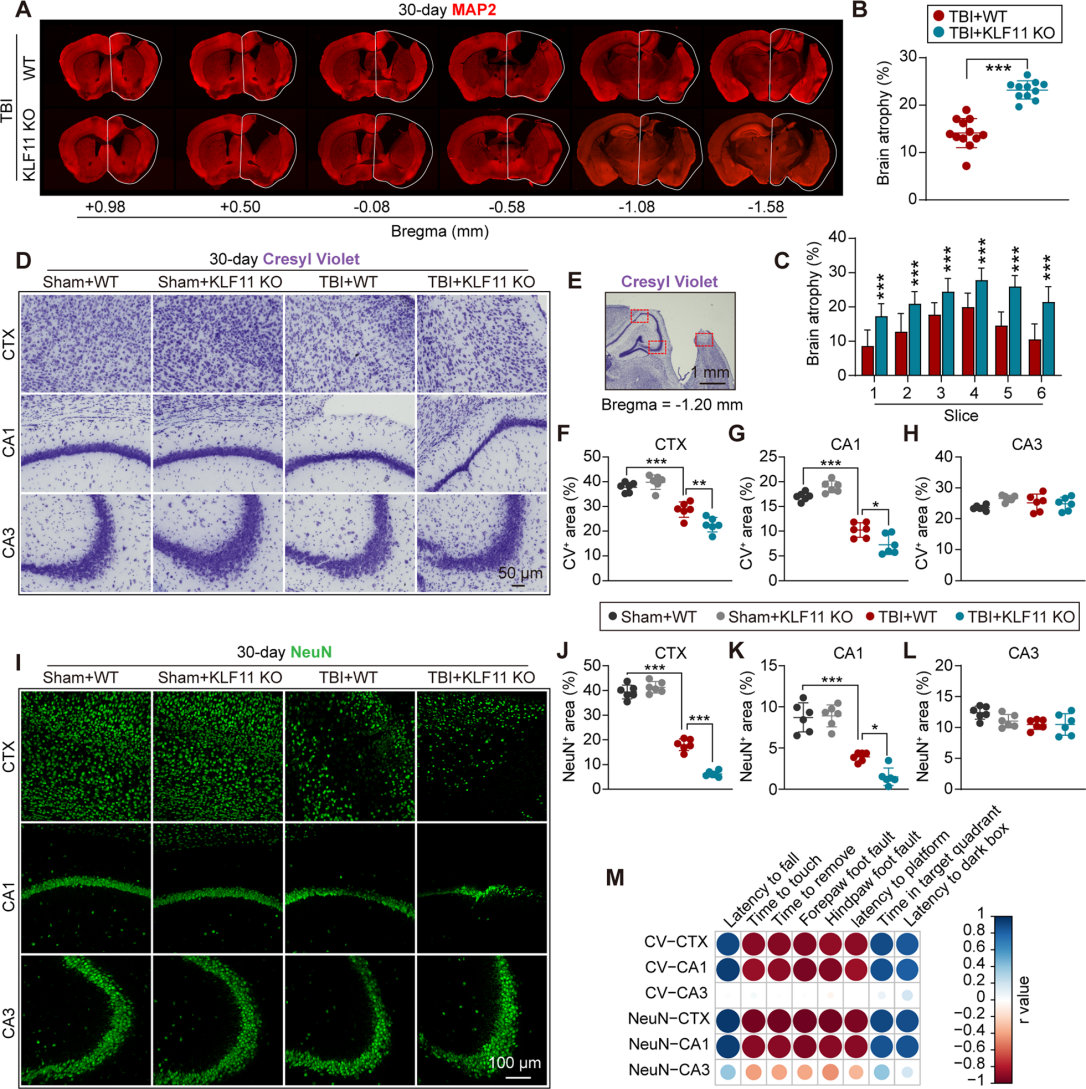
Genetic deletion of KLF11 aggravates brain tissue loss in mice after TBI. KLF11 KO and WT mice were subjected to TBI or sham operation, followed by a 30-d survival period. Tissue loss was examined in brain sections at 30 d after operation by using MAP2 immunostaining. **A** Representative images of MAP2 immunostaining (Bregma from + 0.98 mm to − 1.58 mm). **B**, **C** Quantitative analysis of total volume of brain atrophy and tissue atrophy area in each brain slice (*n* = 11–12/group, unpaired *t*-test). Neuronal loss was examined by Cresyl Violet (CV) staining and NeuN immunostaining in brain sections at 30 d after TBI. **D** Representative images of CV staining in the peri-lesional cerebral cortex (CTX), hippocampal CA1, and hippocampal CA3 regions. E Peri-lesional brain areas (rectangles) in the CTX, CA1, and CA3 where images in D and I were captured. **F**–**H** Quantitative analysis of CV-stained neurons in the peri-lesional CTX, CA1, and CA3 regions (*n* = 6/group, one-way ANOVA with Tukey post hoc test). **I** Representative images of NeuN immunostaining in the peri-lesional CTX, CA1, and CA3 regions. **J**–**L** Quantitative analysis of NeuN-immunopositive neurons in the peri-lesional CTX, CA1, and CA3 regions (*n* = 6/group, one-way ANOVA with Tukey post hoc test). **M** Correlation analysis between sensorimotor or cognitive outcome and CV-stained/NeuN-positive neurons in the peri-lesional CTX, CA1, and CA3 regions (*n* = 6/group, Pearson correlation analysis). Data are presented as mean ± SD. *p < 0.05, **p < 0.01 or ***p < 0.001 versus TBI + WT group

To explore potential mechanisms underlying exacerbated neurobehavioral dysfunction in mice with KLF11 genetic deletion, we next examined the volume of brain tissue loss in KLF11 KO and WT mice 30 d after TBI by using microtubule-associated protein 2 (MAP2, a neuronal marker) immunofluorescence staining (Fig. 3A). In comparison with WT controls, KLF11 KO mice exhibited a larger volume of brain atrophy (Fig. 3B) and larger areas of atrophy in cross-sectional areas (Fig. 3C), suggesting an increased gross tissue loss after TBI.

In parallel, we also examined cortical and hippocampal neuronal loss in KLF11 KO and WT mice 30 d after TBI by using CV histological staining (Fig. 3D–H) and NeuN immunofluorescence staining (Fig. 3I–L). CV (Fig. 3D) and NeuN staining (Fig. 3I) images were taken from the peri-lesional cerebral cortex (CTX), hippocampal CA1, and hippocampal CA3 regions as shown in Fig. 3E. Quantitative results showed that TBI induced significant neuronal loss in the peri-lesional CTX (Fig. 3F, J) and CA1 (Fig. 3G, K) regions, but not in the CA3 region (Fig. 3H, L) compared with sham controls. Even worse, genetic deletion of KLF11 resulted in increased neuronal loss in the peri-lesional CTX (Fig. 3G, K) and CA1 regions, but not CA3 region compared with WT controls (Fig. 3H, L). Additionally, Pearson correlation analyses were conducted to examine correlations between neuronal densities from the cerebral cortex (CTX), hippocampal CA1, and hippocampal CA3 regions and neurological outcomes. As shown in Fig. 3M, neuronal densities in the pre-lesional CTX and CA1 regions were significantly positively correlated with the time to touch/remove adhesive tape, forepaw/hindpaw foot fault rate, and latency to find the platform in neurobehavioral tests. Neuronal densities were significantly negatively correlated with latency to fall, time in the target quadrant, and latency to the dark box. However, neuronal density in the hippocampal CA3 region was not correlated to neurological outcomes (Fig. 3M). These results indicated that neuronal numbers in the peri-lesional CTX and CA1 regions were highly associated with post-trauma sensorimotor and cognitive dysfunctions. Reduced neuronal densities in peri-lesional CTX and CA1 regions may contribute to worsened sensorimotor deficit and cognitive impairment in KLF11 KO mice after TBI compared with WT controls.

### Genetic deletion of KLF11 deteriorates white matter injury in mice after TBI

**Fig. 4:**
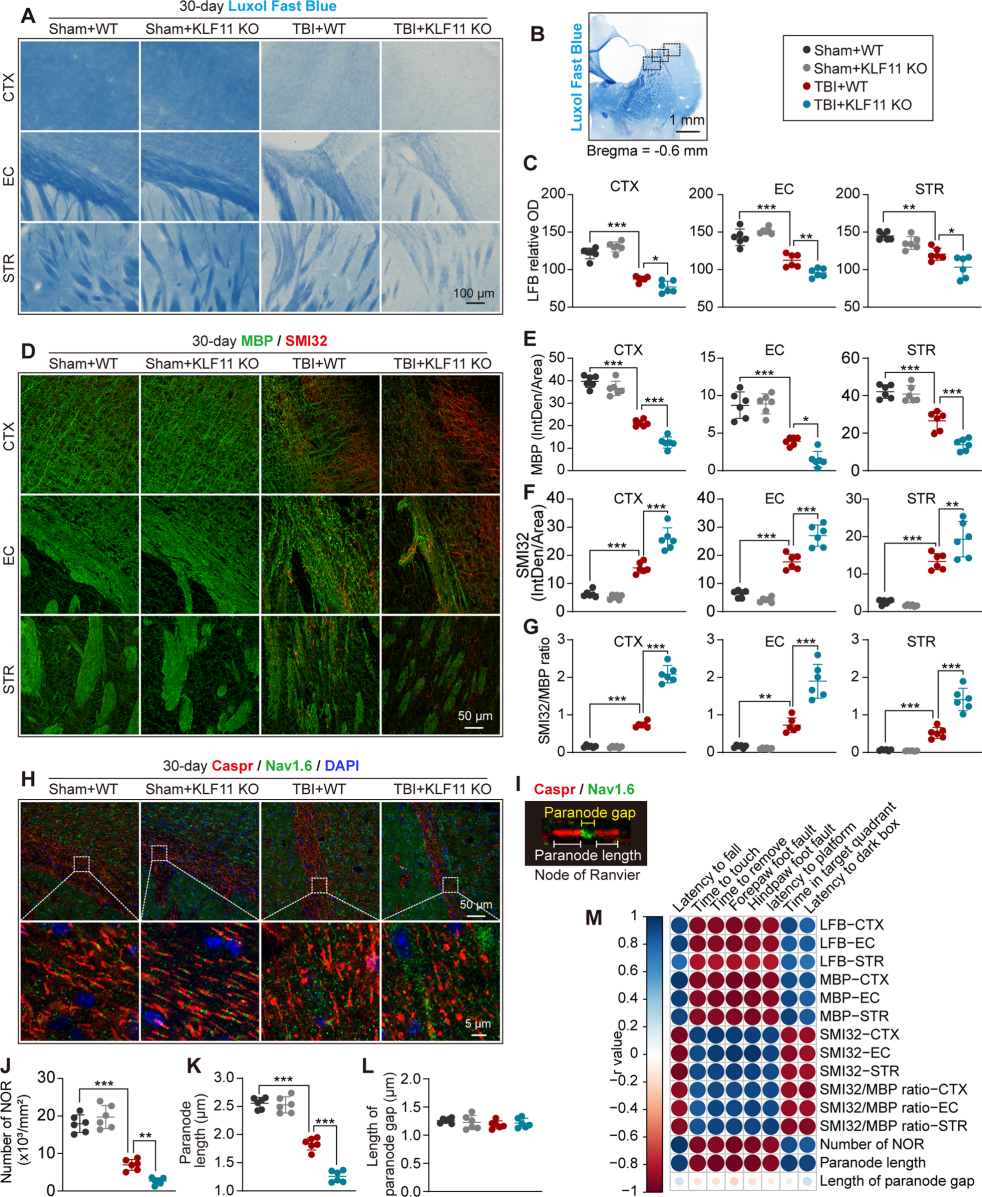
KLF11 genetic deficiency exacerbates white matter injury in mice after TBI. Luxol fast blue (LFB) histological staining and MBP/SMI32 double-immunostaining were applied to detect white matter integrity in KLF11 KO and WT mouse brains at 30 d after TBI. **A** Representative LFB images of the pericontusional CTX, external capsule (EC), and striatum (STR) regions. **B** Pericontusional brain areas (rectangles) in the CTX, EC, and STR where images in **A**, **D**, and **H** were captured. **C** Quantitative analysis of relative OD values of LFB in pericontusional CTX, EC, and STR regions (*n* = 6/group, one-way ANOVA with Tukey post hoc test). **D** Representative images of MBP (green) and SMI32 (red) double-immunostaining in pericontusional CTX, EC, and STR regions. Quantitative analysis of MBP fluorescence intensities (**E**), SMI32 fluorescence intensities (**F**), and the ratio of SMI32/MBP (**G**) in the pericontusional CTX, EC, and STR regions (*n* = 6/group, one-way ANOVA with Tukey post hoc test). The nodes of Ranvier (NOR) were double-immunostained by Caspr/Nav1.6. **H** Representative images of Caspr (Red)/Nav1.6 (green) in the pericontusional EC region (rectangles: enlarged area of low-magnification images). **I** An image showing the composition and normal structure of the nodes of Ranvier. Quantitative analysis of the number of NORs (**J**), paranode length (**K**), and length of paranode gaps (**L**). **M** Correlation analysis of sensorimotor or cognitive outcome and white matter integrity (*n* = 6/group, Pearson correlation analysis). Data are presented as mean ± SD. *p < 0.05, **p < 0.01 or ***p < 0.001 versus TBI + WT group

Demyelination and diffuse axonal damage highly contribute to post-trauma cognitive dysfunction. Therefore, we examined myelin loss and axonal damage by using LFB histological staining (Fig. 4A–C) and myelin basic protein (MBP, myelin marker)/neurofilament heavy polypeptide (clone: SMI32, injured axon marker) double-immunofluorescence staining (Fig. 4D–G) in the peri-lesional CTX and striatum (STR) (Fig. 4B) 30 d after TBI. During brain trauma, WT mice exhibited significant demyelination and axonal damage when compared with sham controls, showing a reduced relative OD value of LFB staining (Fig. 4C), decreased MBP fluorescence intensity (Fig. 4E), increased SMI32 fluorescence intensity (Fig. 4F), and an increased SMI32/MBP ratio (Fig. 4G) in the peri-lesional CTX, EC, and STR regions. Of note, genetic deletion of KLF11 in mice worsened white matter injury in TBI brains compared with WT controls, as shown with further reduction of the LFB OD value and MBP fluorescence intensity, as well as further elevation of SMI32 fluorescence intensity and the SMI32/MBP ratio in the peri-lesional CTX, EC, and STR (Fig. 4C, E–G) regions. We also examined structural integrity of the nodes of Ranvier (NOR) (Fig. 4H–L) by using contactin-associated protein (Caspr)/sodium channel (Nav1.6) double-immunofluorescence staining in the peri-lesional EC region (Fig. 4H, I) in mice 30 d after TBI. We noted that brain trauma elicited a significant reduction in the number of NOR (Fig. 4H, J) and paranode length (Fig. 4K) in mice 30 d after TBI compared with sham controls. Interestingly, genetic deletion of KLF11 in mice exhibited even further reduction in the number of NOR and shorter paranode length than WT controls, implying even worsened function of the NOR (Fig. 4J, K). We failed to observe any significant difference in the length of the paranode gap among experimental groups (Fig. 4L). Moreover, as demonstrated in Fig. 4M and Additional file 4: Tables S3, Additional file 5: Table S4, Pearson correlation analyses showed a positive correlation between white matter structural parameters (LFB, MBP/SMI32, Caspr/Nav1.6) and sensorimotor/cognitive outcomes. The increased post-traumatic white matter injury in KLF11 KO mice may causatively contribute to severer neurobehavioral dysfunction seen versus WT controls after TBI.

### Genetic deletion of KLF11 promotes astrocytic activation in mouse TBI brains

**Fig. 5:**
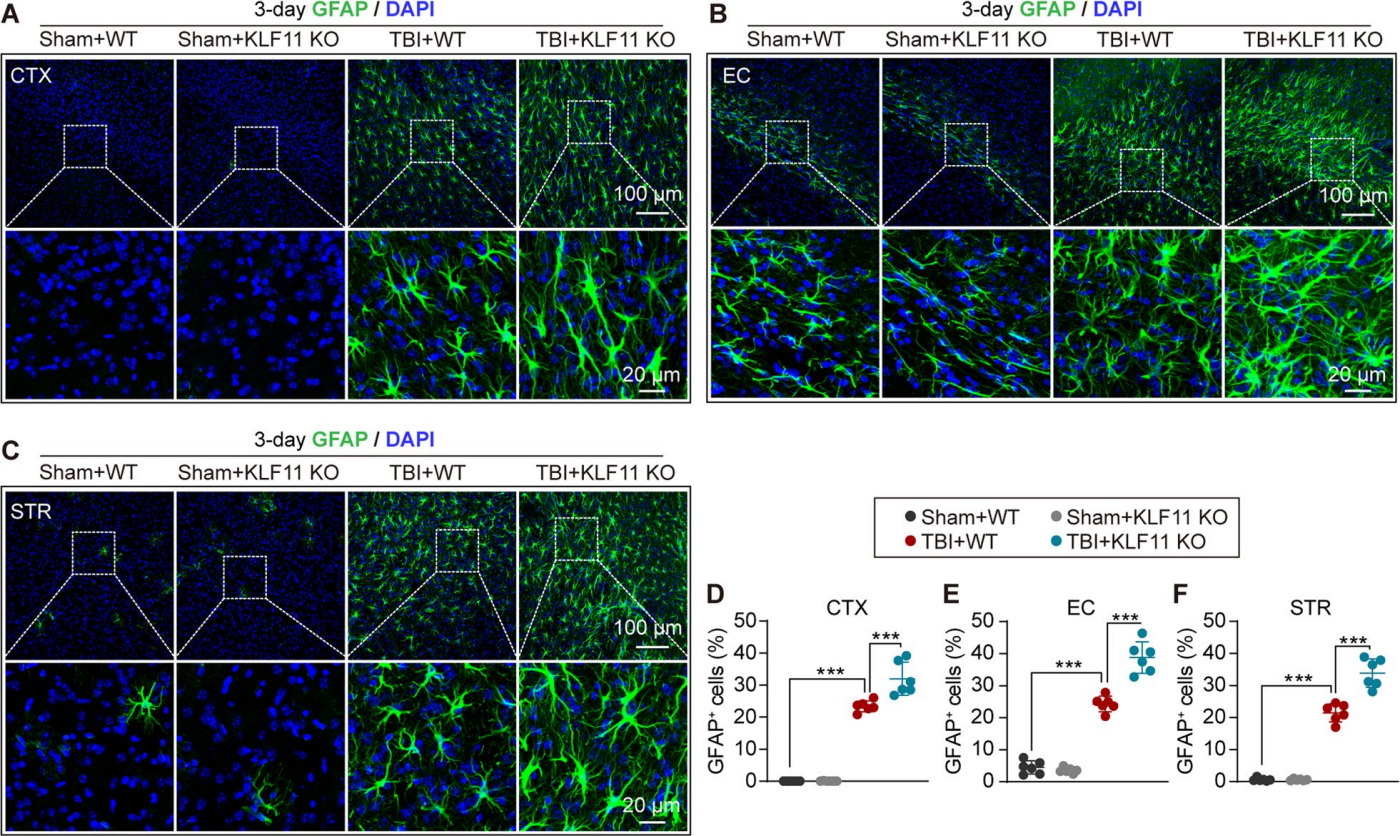
KLF11 genetic deficiency promotes astrocytic activation in the white and grey matter of mice after TBI. KLF11 KO and WT mice were subjected to TBI or sham operation and reactive astrocytes were detected in brain sections at 3 d after surgery by GFAP immunostaining. **A**–**C** Representative images of GFAP (green) and DAPI (blue) in the peri-lesional CTX (**A**), EC (**B**), and STR (**C**) regions (rectangles: enlarged area of low-magnification images). **D**–**F** Quantitative analysis of GFAP-positive astrocytes in the peri-lesional CTX (**D**), EC (**E**), and STR (**F**) regions. Data are presented as mean ± SD, *n* = 6/group. Statistical analyses were performed by one-way ANOVA with the Tukey post hoc test. *p < 0.05, **p < 0.01 or ***p < 0.001 versus TBI + WT group

Excessive and long-lasting astrocytic activation is another widespread pathological change in post-trauma brains. Therefore, we examined astrocytic activation in the pericontusional brains of KLF11 KO and WT mice 3 days after TBI by using glial fibrillary acidic protein (GFAP) immunofluorescence staining. As demonstrated in Fig. 5A–C, KLF11 KO and WT mice exhibited considerable GFAP-positive cells in the CTX, EC, and STR regions, and there were no significant differences in astrocytic activation in KLF11 KO and WT mice under sham conditions. After 3 d of TBI, WT mice exhibited increased GFAP-positive cells in the pericontusional CTX, EC, and STR regions when compared with sham controls, suggesting that TBI induced widespread astrocytic activation in white matter and grey matter (Fig. 5D–F). Genetic deletion of KLF11 in mice further promoted post-trauma astrocytic activation in CTX, EC, and STR brain regions and also showed more GFAP-positive astrocytes in these regions when compared with WT controls (Fig. 5D–F).

### KLF11 genetic deletion promotes the polarization of microglia/macrophages to a pro-inflammatory phenotype in mouse TBI brains

**Fig. 6:**
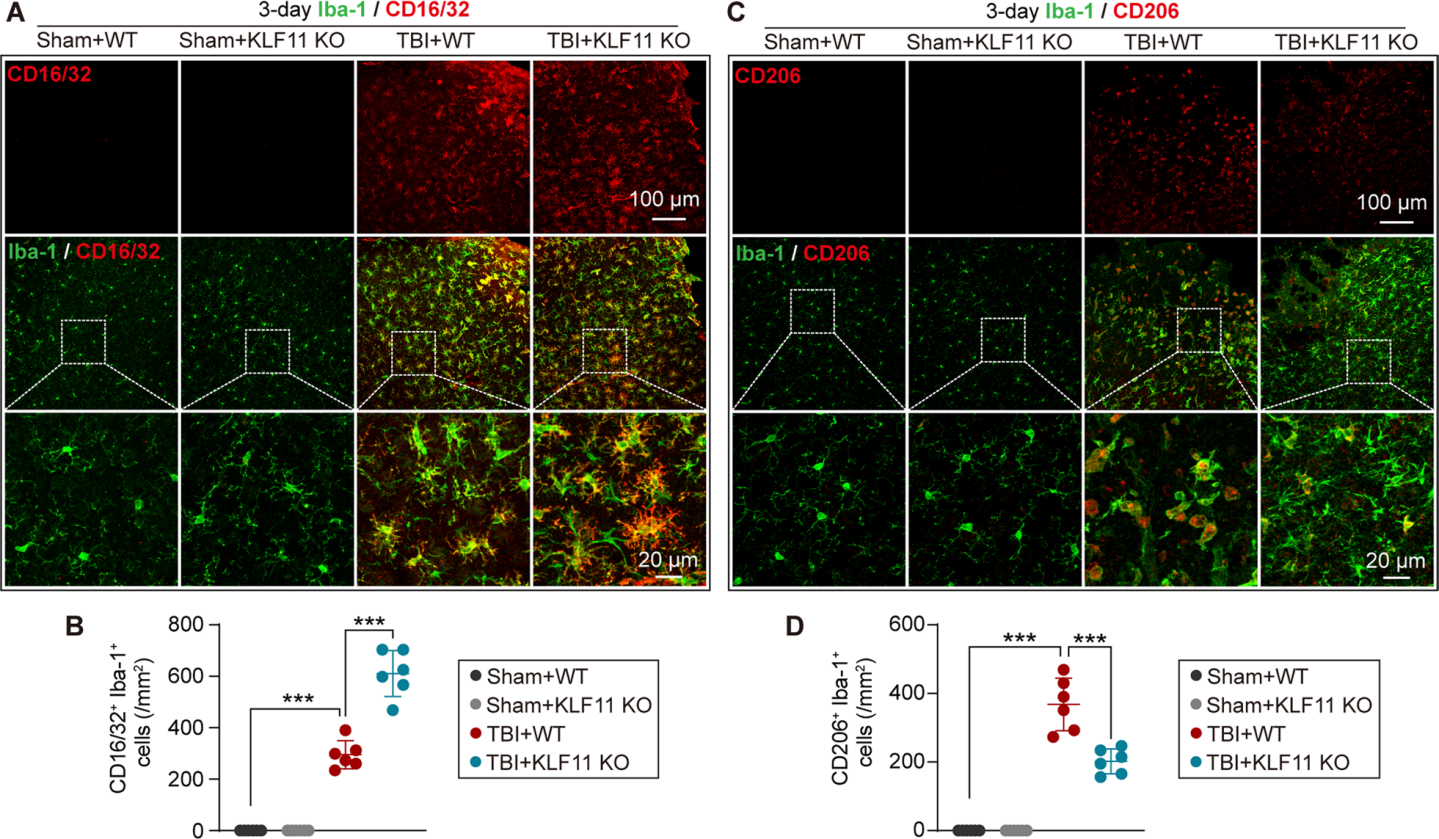
Genetic deletion of KLF11 increases pro-inflammatory microglia/macrophage polarization in mice after TBI. KLF11 KO and WT mice were subjected to TBI or sham operation and the activity of microglial polarization was examined in pericontusional brain regions at 3 d after TBI by double-immunostaining Iba-1 with CD16/32 (M1-phenotype) or CD206 (M2-phenotype). **A** Representative images of Iba-1 (green) and CD16/32 (red) double-immunofluorescence staining (rectangles: enlarged area of low-magnification images). **B** Quantitative analysis of Iba-1 and CD16/32 co-immunostained microglia/macrophages in experimental groups. **C** Representative images of Iba-1 (green) and CD206 (red) double-immunofluorescence staining (rectangles: enlarged area of low-magnification images). **D** Quantitative analysis of Iba-1 and CD206 co-immunostained microglia/macrophages in experimental groups. Data are presented as mean ± SD, *n* = 6/group. Statistical analyses were performed by one-way ANOVA with Tukey’s post hoc test. *p < 0.05, **p < 0.01 or ***p < 0.001 versus TBI + WT group

Next, we examined the effect of KLF11 genetic deletion on microglia/macrophage polarization by co-immunostaining Iba-1 with either CD16/32 (an M1-phenotype microglia/macrophage marker) or CD206 (an M2-phenotype microglia/macrophage marker) in mouse brain sections at 3 d after TBI (Fig. 6A, C). After 3 d of TBI, WT mice showed increases in both M1- (Iba-1&CD16/32-positive) and M2-phenotype (Iba-1&CD206-positive) microglia/macrophages in peri-lesional brain regions when compared with sham controls (Fig. 6B, D). However, genetic deletion of KLF11 significantly increased pro-inflammatory CD16/32-positive microglia/macrophages but decreased inflammatory-resolving CD206-positive microglia/macrophages in peri-lesional brain regions 3 d after TBI when compared with WT controls (Fig. 6B, D). These data suggest that KLF11 genetic deletion in mice promotes microglia/macrophage polarization to a pro-inflammatory M1-phenotype in response to brain trauma.

### KLF11 genetic deletion increases the infiltration of peripheral neutrophils and macrophages in mouse brains after TBI

**Fig. 7:**
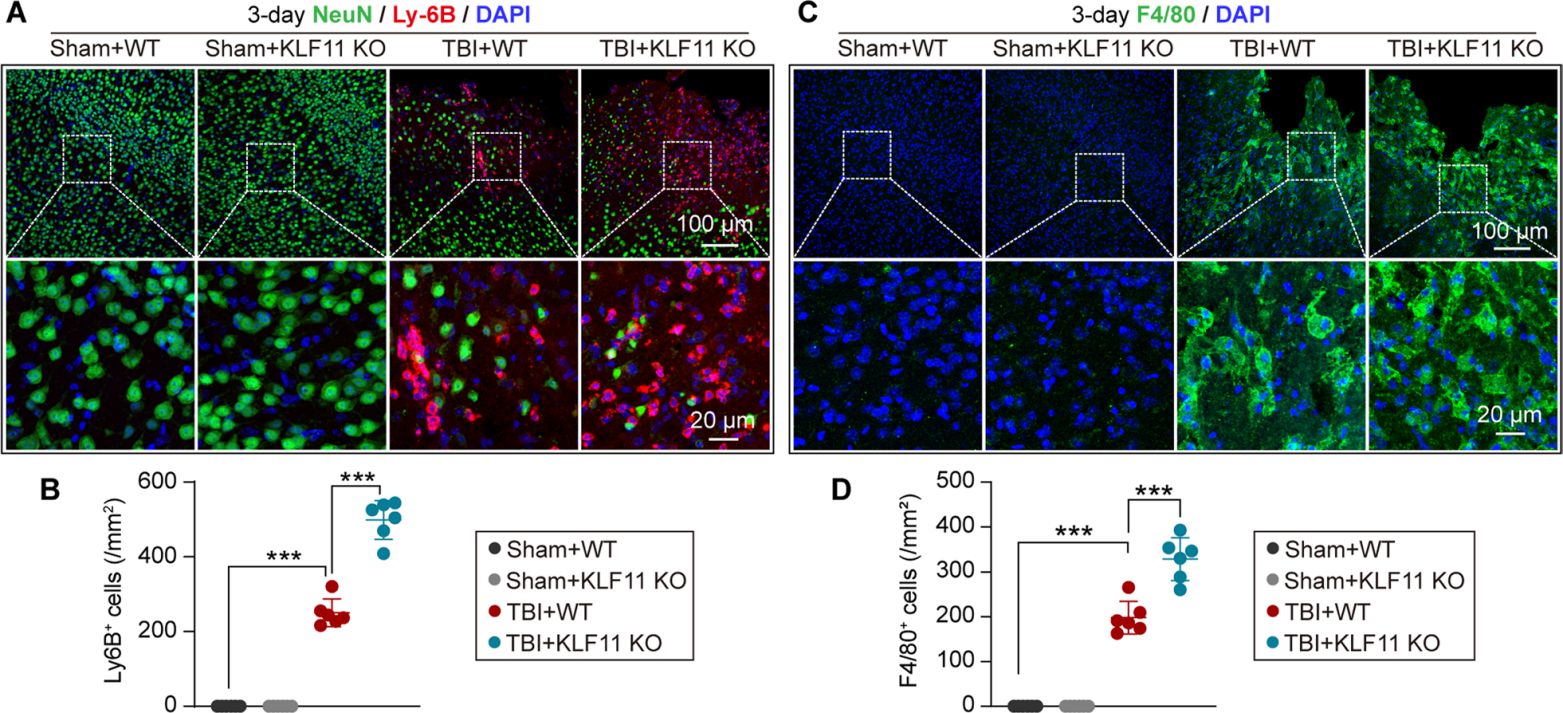
KLF11 genetic deletion increases TBI-induced infiltration of peripheral neutrophils and macrophages. KLF11 KO and WT mice were subjected to TBI or sham operation and the peripheral infiltration of neutrophils and macrophages was assessed by double-immunostaining in brain sections 3 d after operation. **A** Representative images of NeuN (green), Ly-6B (red, a marker of neutrophils), and DAPI (blue) immunostaining in peri-lesional brain regions. Rectangles: enlarged area of low-magnification images. **B** Quantitative analysis of Ly-6B-positive neutrophils from peri-lesional brains or sham mouse brains. **C** Representative images of F4/80 (green) and DAPI (blue) immunostaining in peri-lesional brain regions. Rectangles: enlarged area of low-magnification images. **D** Quantitative analysis of F4/80-positive macrophages/microglia from peri-lesional or sham mouse brains. Data are presented as mean ± SD, *n* = 6/group. Statistical analyses were performed by one-way ANOVA with Tukey’s post hoc test. *p < 0.05, **p < 0.01 or ***p < 0.001 versus TBI + WT group

Post-trauma BBB disruption and leakage result in several subsequent pathological events, including the infiltration of peripheral immune cells such as neutrophils and macrophages into the parenchyma. Hence, the peripheral infiltration of neutrophils and macrophages was examined in the brains of KLF11 KO and WT mice 3 d after TBI by using Ly-6B (a neutrophil marker) and F4/80 (a marker for macrophages and microglia) immunostaining. After 3 days of TBI, WT mice exhibited increased Ly-6B-positive neutrophils and F4/80-positive macrophages/microglia in pericontusional brain regions versus the sham controls (Fig. 7A, C). Even worse, genetic deletion of KLF11 further increased the infiltration of neutrophils and macrophages/microglia to post-trauma brains, showing more neutrophils and macrophages/microglia in the pericontusional brain regions when compared with WT controls (Fig. 7B, D). These data suggest that genetic deletion of KLF11 increased the infiltration of blood-borne immune cells to enhance cerebral inflammatory responses in mice after TBI.

### Genetic deletion of KLF11 enhances brain inflammatory mediators in mice after TBI

**Fig. 8:**
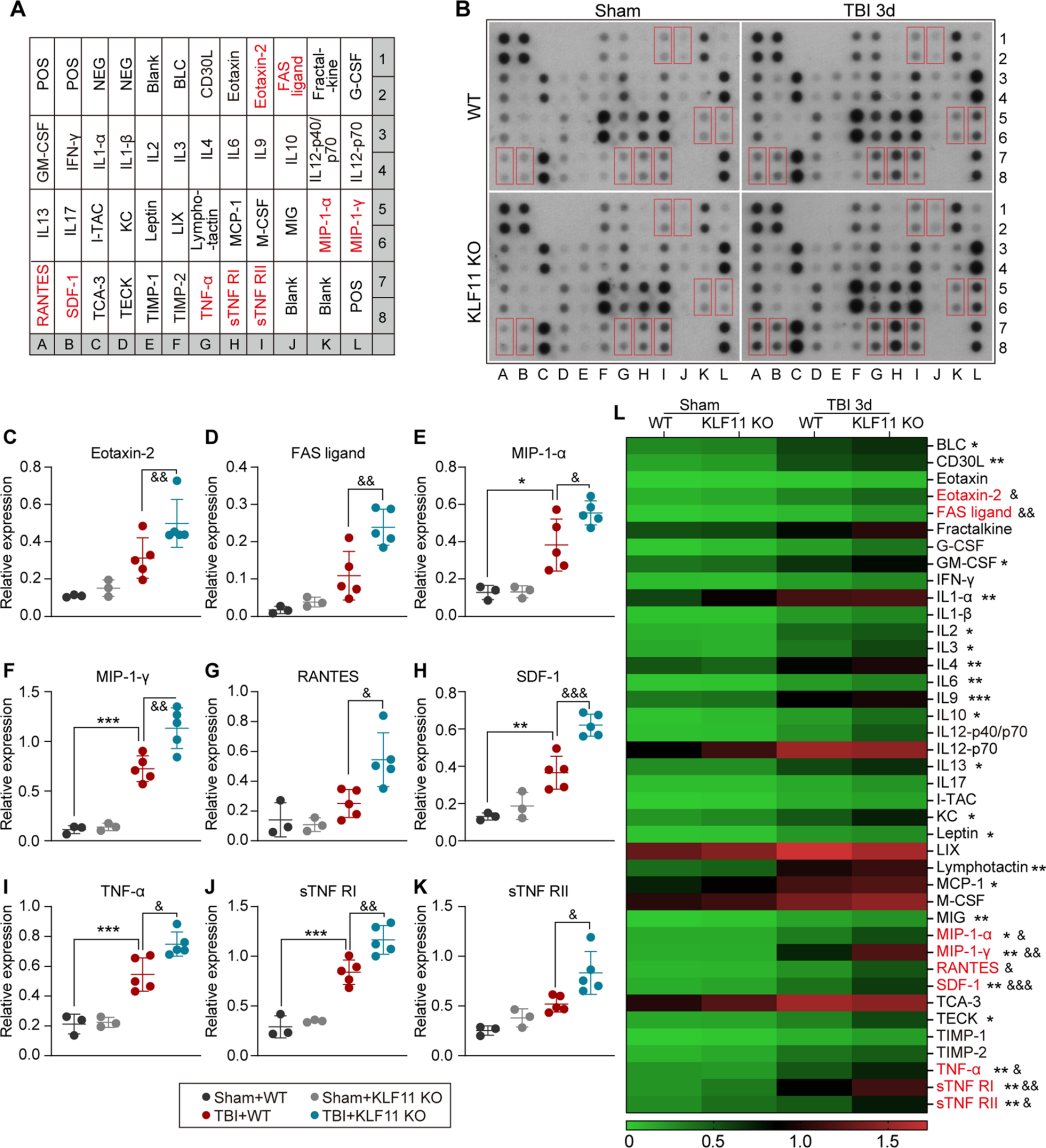
KLF11 genetic deficiency in mice increases the inflammatory burden in post-trauma brains. KLF11 KO and WT mice were subjected to TBI or sham operation and a panel of 40 inflammatory mediators was measured in mouse brains at 3 d after CCI operation. **A** Array map of 40 inflammatory mediators. **B** Representative blots of the inflammatory array in experimental groups. **C**–**K** Quantitative analysis showed significantly increased inflammatory mediators in the brains of KLF11 KO mice in comparison with WT controls 3 d after TBI, including Eotaxin-2, FAS ligand, MIP-1-α, MIP-1-γ, RANTES, SDF-1, TNF-α, sTNF RI, and sTNF-RII. Data are presented as mean ± SD, *n* = 3–5/group. Statistical analyses were performed by one-way ANOVA with Tukey post hoc test. **L** A heatmap showing the mean expression levels of 40 brain inflammatory mediators in experimental groups. *p < 0.05, **p < 0.01 or ***p < 0.001 Sham + WT versus TBI + WT group. ^&^*p* < 0.05, ^&&^*p* < 0.01 or ^&&&^*p* < 0.001 TBI + KO versus TBI + WT group

We further profiled the potential target inflammatory cytokines underlying the aggravated pathological and functional outcomes in TBI mice with KLF11 genetic deficiency. Experimental TBI was induced in both KLF11 KO and WT mice and a panel of 40 inflammatory mediators was analyzed in post-trauma brains 3 days after TBI by using an inflammatory antibody array kit. The distribution of 40 inflammatory mediators and representative immunoblots are shown in Fig. 8A, B. Our data demonstrated that 23 inflammatory mediators including BLC, CD30L, GM-CSF, IL-1α, IL-1β, IL-2, IL-3, IL-4, IL-6, IL-9, IL-10, IL-13, KC, leptin, lymphotactin, MCP-1, MIG, MIP-1α, MIP-1γ, SDF-1, TNF-α, sTNF RI, and sTNF RII were significant elevated in peri-lesional brain regions at 3 days after TBI when compared with sham controls (Fig. 8L). Interestingly, genetic deletion of KLF11 further increased the protein levels of 9 pro-inflammatory mediators including eotaxin-2, FAS ligand, MIP-1α, MIP-1γ, RANTES, SDF-1, TNF-α, sTNF RI, and sTNF RII in peri-lesional brain regions at 3 d after TBI (Fig. 8C–K). These data imply that the boosted pro-inflammatory mediators found in the post-trauma brains of KLF11 KO mice might be downstream targets of KLF11 and these pro-inflammatory mediators contribute to the aggravated neurological dysfunction after TBI.

## Discussion

Krüppel-like factors play a critical role in neurological diseases, including neurodegenerative diseases, ischemic stroke, epilepsy, CNS carcinoma, stress, depression, alcoholism, neuroinflammation, and schizophrenia [16, 29–31]. However, the functional importance of Krüppel-like factors in the pathogenesis of brain trauma has been less explored. The present study is the first to characterize the impact of KLF11 genetic deficiency on traumatic brain injury. Genetic deletion of KLF11 aggravated long-term sensorimotor and cognitive dysfunctions in mice after TBI, which was positively correlated with increased brain tissue or neuronal loss and increased white matter injury in post-trauma brains. Genetic deletion of KLF11 further promoted activation of astrocytes and polarization of pro-inflammatory microglia/macrophages, as well as increased the infiltration of peripheral neutrophils and macrophages to brain parenchyma in TBI mice. Also, genetic deletion of KLF11 in mice boosted the release of inflammatory mediators to the post-trauma brains. Our data indicate that KLF11 plays a protective role in the pathogenesis of brain trauma.

Cumulative studies have shown that neurobehavioral dysfunction and sequelae are very common in TBI patients [1, 10, 32] as well as in rodent experimental TBI models [21, 33, 34]. We have previously reported that genetic deletion of KLF11 in mice delayed the recovery of sensorimotor function at 7 d of ischemic stroke [18]. We have also recently shown that endothelium-targeted transgenic overexpression of KLF11 in mice improved sensorimotor deficits at the acute phase of ischemic stroke [19]. Until now, the effect of KLF11 on post-traumatic neurologic deficits and recovery is not well understood. In this study, we demonstrated that genetic deletion of KLF11 aggravated long-term sensorimotor deficits and learning/memory impairments following TBI, suggesting a critical role of KLF11 in the regulation of both sensorimotor and cognitive functions after brain trauma.

The initial impact of TBI and long-lasting secondary damage contribute to the brain tissue loss and neuronal death [4], which results in entire brain atrophy or focal atrophy in specific brain regions of patients with brain trauma [35, 36]. Animal studies also showed that experimental TBI elicits continuous brain tissue loss and neuronal degeneration in the pericontusional cerebral cortex and hippocampus [34, 37]. Previously, several studies have demonstrated that KLF family members are able to regulate axonal regeneration and neuronal apoptosis [38, 39]. For instance, Li et al. showed that intraventricular administration of AAV-KLF7 upregulated the JAK2/STAT3 pathway and attenuated hippocampal neuronal apoptotic activity in mice after TBI [17]. Dobrivojevic et al. also found that KLF8 is strongly expressed throughout adult mouse brains including the cerebral cortex, hippocampus, striatum, and other regions although the function of KLF8 remains to be determined [40]. We previously found that genetic deletion of KLF11 in mice increased brain infarction, while endothelium-selected transgenic overexpression of KLF11 reduced the cerebral infarct volume after ischemic stroke [18, 41]. Up to now, KLF11 regulation of brain tissue loss and atrophy is unclear. In this study, we are the first to demonstrate that genetic deletion of KLF11 further worsened TBI-induced brain atrophy and neuronal death in the cerebral cortex and hippocampal CA1 regions in mice after TBI. Moreover, we also noted that the reduced neuronal densities in the pericontusional cerebral cortex and CA1 regions were positively correlated with sensorimotor and cognitive dysfunctions in mice after TBI. Our data suggest KLF11 as a novel neuroprotective mediator in TBI.

Chronic white matter injury contributes to long-term cognitive impairment following TBI [42]. Kinnunen et al. demonstrated the fornix was correlated with associative learning and memory, whereas frontal lobe connections were correlated with executive function in TBI patients [5]. In experimental TBI models, slowed and failed signal conduction along with damage to the structural and molecular compositions of myelinated axons were also identified in white matter of mice after TBI [43]. It was reported that white matter injury-modifying therapies such as tissue plasminogen activator and interleukin-4 can effectively promote functional recovery in TBI [34, 37]. KLFs can regulate axonal structures and function. For example, Avila-Mendoza et al. showed that KLF9 and KLF13 inhibit neurite/axon growth in hippocampal neurons partially by inhibiting the cAMP signaling pathway [44]. In addition, Galvao et al. revealed that inhibition of the KLF9–Dusp14 pathway increased axonal growth in vitro and promoted survival and optic nerve regeneration after optic nerve injury in vivo, suggesting modulation of KLF9 as a potential therapeutic approach to reducing axonal damage in neurological diseases. Moreover, KLFs also actively take part in the myelination process. Laitman et al. demonstrated that KLF6 acted as a key coordinator of gp130-Stat3 signaling and importin-based control of nuclear trafficking, which is essential for myelination in the brain [45]. Less attention has been paid to the effects of KLFs on white matter damage in brain trauma. In this study, we found that genetic deletion of KLF11 significantly increased axonal degeneration in mice after TBI. Moreover, KLF11 genetic deficiency also accelerated demyelination activities, including myelin loss and structural abnormality of the nodes of Ranvier in mouse brains after TBI. Of importance, myelin loss and axonal damage had a strong positive correlation with sensorimotor and cognitive deficits in KLF11 KO mice following TBI. Our data highlight the essential role of KLF11 in maintaining white matter integrity in response to brain trauma.

Acute brain inflammatory responses act as key pathological characteristics in the pathogenesis of TBI. TBI triggers a rapid astrocytic response to brain trauma [12, 46–49]. Although an increased number of astrocytes in post-trauma brains have been shown to seal the disrupted BBB to a certain extent [50], an excessive increase in the number of astrocytes may lead to an overabundance of pro-inflammatory responses that result in axonal injury, vascular disruption, and local ischemia in post-traumatic brains [47, 49]. Previous studies have demonstrated that high levels of serum GFAP and S100B (an astrocyte marker) may function as predictors for increased mortality and unfavorable outcomes in TBI [48, 49]. Park et al. demonstrated that astroglial KLF4 expression was induced within 3 days and persisted for at least 4 weeks following ischemic stroke in mice, indicating that KLF4 was associated with the post-stroke astrocytic reaction [51]. Our present study is the first to report that genetic deletion of KLF11 in mice promoted the activation of astrocytes in both white and grey matter after TBI. However, the underlying mechanisms by which KLF11 genetic deficiency activates cerebral astrocytes remain to be further investigated in brain trauma.

Microglia/macrophage polarization exerts both beneficial (M2-phenotype) and detrimental (M1-phenotype) roles in TBI [12]. Previous studies in rodent CCI models indicated that both M1-like (e.g., CD16/32) and M2-like (e.g., CD206) microglial cells were increased in pericontusional brain regions after TBI [12]. CD16/32 is an Fc receptor that mediates phagocytosis [52]. The expression of CD206 on microglia mediates an anti-inflammatory response by facilitating the recognition, binding, and endocytosis of pathogens [52]. Previous studies have demonstrated that CD16/32-expressing microglial cells promote a pro-inflammatory response, while CD206-expressing microglial cells mediate an anti-inflammatory response in several neurological diseases, such as ischemic stroke and brain trauma [22, 53, 54]. A few studies have previously explored the role of Krüppel-like factors in the regulation of microglia/macrophage polarization. For instance, Liao et al. demonstrated that KLF4 was robustly induced in M2-like microglia/macrophages but largely reduced in M1-like microglia/macrophages in human inflammatory paradigms in vivo [55]. Additionally, microglia/macrophages with loss-of-KLF4 function exhibited increased pro-inflammatory gene expression after LPS stimuli [55]. The role of KLF family members, including KLF11, in the polarization of microglia/macrophages after TBI is poorly explored. Here, we reported for the first time that genetic deletion of KLF11 promoted the polarization of resting microglia/macrophages to CD16/32-expressing but not to CD206-expressing microglia/macrophages in mice 3 days after experimental TBI. Thus, our observations reveal KLF11 as a novel anti-inflammatory mediator via its regulation of microglia/macrophage polarization in traumatic brains.

Post-traumatic BBB disruption and leakage trigger peripheral immune cells, including neutrophils and macrophages, to be recruited to pericontusional brain regions within hours and lasting for several days after TBI. KLFs are involved in regulating BBB integrity in ischemic stroke and endothelial function in abdominal aortic aneurysm [19, 20, 56, 57]. We have previously demonstrated that endothelium-targeted transgenic overexpression of KLF11 promotes expression of the endothelial tight junction protein, ZO-1 and decreased BBB leakage and brain neuroinflammation in experimental ischemic stroke [19]. In another study, we also reported that KLF11 serves as a co-regulator of PPARγ to protect mice against ischemia-induced cerebrovascular injury [20]. In the present study, we extended our previous findings on the KLF11 role in ischemic stroke and found that genetic deletion of KLF11 promotes infiltration of peripheral neutrophils and macrophages to the brain parenchyma in mice after brain trauma, implying that KLF11 genetic deficiency may cause BBB structural damage and functional disorders, which needs to be further investigated.

Along with the activation of glial cells and infiltration of peripheral immune cells, an abundance of inflammatory mediators are released into the cerebral parenchyma in response to brain trauma stimuli [9, 11, 13, 58]. Previous studies have highlighted the important role of KLFs in regulating inflammatory responses [16, 59]. For example, KLF4 gene expression in macrophages was induced by tumor necrosis factor-alpha (TNF-α), interleukin-1β (IL-1β), IFN-γ, LPS and promoted iNOS expression by interacting with NF-kB p65 [60, 61]. Overexpression of KLF4 in vascular endothelial cells provided an anti-inflammatory response, whereas knockdown of KLF4 led to a pro-inflammatory response by enhancing TNF-α and vascular cell adhesion molecule-1 (VCAM-1) expression [62]. It was reported that another KLF family member, KLF2, protected against ischemic stroke-induced BBB disruption and inhibited subsequent brain inflammation [57]. KLF10 and KLF11 were also documented to repress IL-12 subunit p40 production and regulate the function of macrophages [41]. Moreover, Zhao et al. demonstrated that KLF11 genetic deficiency in vascular endothelial cells aggravated abdominal aortic aneurysm formation and KLF11 transgenic overexpression diminished inflammation in the peripheral vascular wall by targeting MMP-9 expression [56]. We have previously reported that genetic deletion of KLF11 enhanced the expression of pro-inflammatory factors IL-6, TNF-α, ICAM-1, and MCP-1 in ischemic brains [18], whereas transgenic overexpression of KLF11 in vascular endothelial cells strongly repressed the expression of TNF-α, IL-1β, MCP-1, IL-6, ICAM-1, and P-selectin in mouse brains after cerebral ischemia [19]. Currently, there are no reported studies on the role of KLFs in regulating cerebral inflammatory mediators following brain trauma. In the present study, we showed that genetic deletion of KLF11 in mice significantly augmented the release of pro-inflammatory cytokines including Eotaxin-2, FAS ligand, MIP-1α, MIP-1γ, RANTES, SDF-1, TNF-α, sTNF RI, and sTNF RII to cerebral parenchyma in response to TBI. Collectively, these data suggest that KLF11 genetic deficiency in mice enhanced cerebral inflammatory burdens in post-trauma brains, and KLF11 regulation of cerebral inflammation may represent one of the major mechanisms for its protective role in brain trauma.

## Conclusion

In conclusion, we demonstrated that KLF11 genetic deficiency worsened long-term sensorimotor and cognitive dysfunctions and also white and grey matter injuries. Next, KLF11 deletion increased astrocytic and microglial activation, infiltration of peripheral neutrophils and macrophages, and the release of cerebral inflammatory cytokines in mice after TBI. These findings suggest that KLF11 acts as a novel brain protective factor in traumatic brain injury. Elucidating the functional importance of KLF11 in brain trauma may lead us to discover novel pharmacological targets for the development of effective therapies against TBI.

## Supplementary Information

**Additional file 1: Figure S1.** Schematic diagram of experimental design. KLF11 KO or WT mice were subjected to TBI or sham operation. Long-term sensorimotor function was tested before and up to 28 days after operation (rotarod test, adhesive tape removal test, and foot fault test). Cognitive function was examined by the Morris water maze test and passive avoidance test in mice 23–30 days after operation. White matter injury was evaluated by histological staining of LFB, double-immunostaining of MBP/SMI32, and Caspr/Nav1.6 in mouse brain sections at 30 d after operation. Grey matter injury was evaluated by histological staining of CV and immunostaining of MAP2 and NeuN. Neuroinflammation was examined by Ly-6B, F4/80, GFAP, Iba-1/CD16/32, and Iba-1/CD206 immunostaining and an inflammatory array.

**Additional file 2: Table S1.** Animal number and mortality rate in this study.

**Additional file 3: Table S2.** List of primary antibodies used in this study.

**Additional file 4: Table S3.** Pearson correlation analysis (*r* value).

**Additional file 5: Table S4.** Pearson correlation analysis (*p* value).

## Data Availability

The datasets used and/or analyzed during the current study are available from the corresponding author upon reasonable request.

## Abbreviations

BBB: Blood–brain barrier
Caspr: Contactin-associated protein
CBF: Cerebral blood flow
CCI: Controlled cortical impact
MWM: Morris water maze
CTX: Cerebral cortex
CV: Cresyl violet
EC: External capsule
GFAP: Glial fibrillary acidic protein
KLF: Krüppel-like factors
KO: Knockout
LFB: Luxol fast blue
MAP2: Microtubule-associated protein 2
MBP: Myelin basic protein
Nav1.6: Sodium channel
NOR: Node of Ranvier
OGD: Oxygen–glucose deprivation
PPARγ: Peroxisome proliferator-activated receptor gamma
SMI32: Neurofilament heavy polypeptide clone: SMI32
STR: Striatum
TBI: Traumatic brain injury
WT: Wild-type
