# Supplementary figures and images for: Genetic deletion of Krüppel-like factor 11 aggravates traumatic brain injury
Source: J Neuroinflammation. 2022 Nov 19;19:281. doi: 10.1186/s12974-022-02638-0 (PMC9675068; doi:10.1186/s12974-022-02638-0)

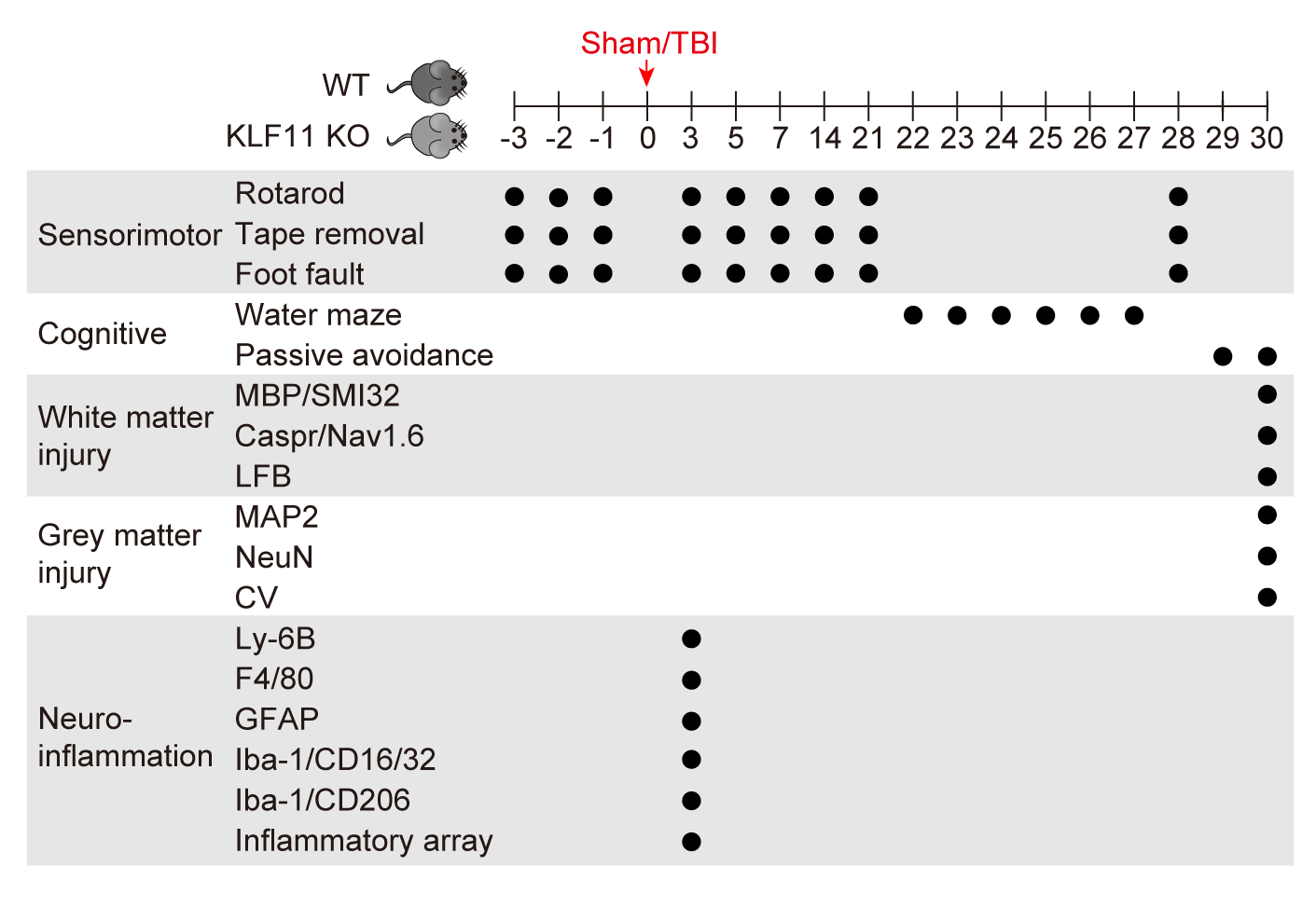

Supplement: Supplementary file 1 — Additional file 1: Figure S1. Schematic diagram of experimental design. KLF11 KO or WT mice were subjected to TBI or sham operation. Long-term sensorimotor function was tested before and up to 28 days after operation (rotarod test, adhesive tape removal test, and foot fault test). Cognitive function was examined by the Morris water maze test and passive avoidance test in mice 23–30 days after operation. White matter injury was evaluated by histological staining of LFB, double-immunostaining of MBP/SMI32, and Caspr/Nav1.6 in mouse brain sections at 30 d after operation. Grey matter injury was evaluated by histological staining of CV and immunostaining of MAP2 and NeuN. Neuroinflammation was examined by Ly-6B, F4/80, GFAP, Iba-1/CD16/32, and Iba-1/CD206 immunostaining and an inflammatory array. [file 12974_2022_2638_MOESM1_ESM.tif]
