# Supplementary material for: Genetic deletion of Krüppel-like factor 11 aggravates traumatic brain injury
Source: J Neuroinflammation. 2022 Nov 19;19:281. doi: 10.1186/s12974-022-02638-0 (PMC9675068; doi:10.1186/s12974-022-02638-0)
Supplement: Supplementary file 2 — Additional file 2: Table S1. Animal number and mortality rate in this study. [file 12974_2022_2638_MOESM2_ESM.docx]

**Table S1. Animal numbers and mortality rates in this study**

| **Endpoint** | **Group** | **Survival** | **Total** | **Experiment** | **Mortality (%)** |
| --- | --- | --- | --- | --- | --- |
| 3-day | Sham+WT | 3 | 3 | Inflammatory array | 0.00 |
|  | Sham+KLF11 KO | 3 | 3 |  | 0.00 |
|  | TBI+WT | 5 | 5 |  | 0.00 |
|  | TBI+KLF11 KO | 5 | 5 |  | 0.00 |
|  | Sham+WT | 6 | 6 | IF stain: GFAP, Iba-1, CD16/32, CD206, Ly6B, NeuN, F4/80 | 0.00 |
|  | Sham+KLF11 KO | 6 | 6 |  | 0.00 |
|  | TBI+WT | 6 | 6 |  | 0.00 |
|  | TBI+KLF11 KO | 6 | 6 |  | 0.00 |
| 30-day | Sham+WT | 12 | 12 | Neurobehavioral tests.  IF&IHC stain (n=6/group): MBP, SMI32, Caspr, Nav1.6, MAP2, NeuN, CV, LFB | 0.00 |
|  | Sham+KLF11 KO | 12 | 12 |  | 0.00 |
|  | TBI+WT | 12 | 12 |  | 0.00 |
|  | TBI+KLF11 KO | 12 | 11 |  | 8.33 |
|  | Total | 88 | 87 |  |  |
