# Supplementary material for: Genetic deletion of Krüppel-like factor 11 aggravates traumatic brain injury
Source: J Neuroinflammation. 2022 Nov 19;19:281. doi: 10.1186/s12974-022-02638-0 (PMC9675068; doi:10.1186/s12974-022-02638-0)
Supplement: Supplementary file 3 — Additional file 3: Table S2. List of primary antibodies used in this study. [file 12974_2022_2638_MOESM3_ESM.docx]

**Table S2. List of primary antibodies used in this study**

| Antibody | Host species | Dilution | Company | Cat # |
| --- | --- | --- | --- | --- |
| MAP2 | Rabbit | 1:500 | Millipore | AB5622 |
| NeuN | Rabbit | 1:500 | EMD Millipore | ABN78 |
| MBP | Rabbit | 1:500 | Abcam | Ab40390 |
| SMI32 | Mouse | 1:500 | BioLegend | 801701 |
| Caspr | Mouse | 1:200 | EMD Millipore | MABN69 |
| Nav1.6 | Rabbit | 1:200 | Millipore | AB5580-50UL |
| GFAP | Rabbit | 1:500 | Sigma-Aldrich | G9269 |
| Iba-1 | Rabbit | 1:500 | Wako | 019-19741 |
| CD16/32 | Mouse | 1:200 | BD Biosciences | 553124 |
| CD206 | Goat | 1:200 | R&D Systems | AF2535 |
| Ly-6B | Rat | 1:100 | Abcam | Ab53453 |
| F4/80 | Mouse | 1:200 | BioLegend | 123102 |
