# Supplementary material for: Genetic deletion of Krüppel-like factor 11 aggravates traumatic brain injury
Source: J Neuroinflammation. 2022 Nov 19;19:281. doi: 10.1186/s12974-022-02638-0 (PMC9675068; doi:10.1186/s12974-022-02638-0)
Supplement: Supplementary file 4 — Additional file 4: Table S3. Pearson correlation analysis (r value). [file 12974_2022_2638_MOESM4_ESM.docx]

**Table S3. Pearson correlation analysis (r value)**

|  | Latency to fall | Time to touch | Time to remove | Forepaw foot fault | Hindpaw foot fault | Latency to platform | Time in target quadrant | Latency to dark box |
| --- | --- | --- | --- | --- | --- | --- | --- | --- |
| LFB-CTX | 0.90 | -0.91 | -0.91 | -0.93 | -0.91 | -0.88 | 0.90 | 0.81 |
| LFB-EC | 0.92 | -0.91 | -0.90 | -0.93 | -0.89 | -0.88 | 0.83 | 0.83 |
| LFB-STR | 0.77 | -0.82 | -0.81 | -0.82 | -0.79 | -0.80 | 0.82 | 0.74 |
| MBP-CTX | 0.96 | -0.92 | -0.92 | -0.95 | -0.95 | -0.91 | 0.88 | 0.86 |
| MBP-EC | 0.93 | -0.90 | -0.94 | -0.95 | -0.94 | -0.91 | 0.90 | 0.82 |
| MBP-STR | 0.91 | -0.90 | -0.91 | -0.92 | -0.94 | -0.89 | 0.92 | 0.84 |
| SMI32-CTX | -0.93 | 0.92 | 0.95 | 0.94 | 0.93 | 0.91 | -0.87 | -0.86 |
| SMI32-EC | -0.93 | 0.89 | 0.94 | 0.96 | 0.97 | 0.91 | -0.90 | -0.85 |
| SMI32-STR | -0.95 | 0.89 | 0.91 | 0.94 | 0.92 | 0.89 | -0.86 | -0.85 |
| SMI32/MBP-CTX | -0.89 | 0.85 | 0.90 | 0.89 | 0.90 | 0.89 | -0.87 | -0.92 |
| SMI32/MBP-EC | -0.87 | 0.83 | 0.91 | 0.90 | 0.92 | 0.89 | -0.87 | -0.85 |
| SMI32/MBP-STR | -0.90 | 0.86 | 0.91 | 0.91 | 0.91 | 0.93 | -0.90 | -0.91 |
| Number of NOR | 0.96 | -0.92 | -0.91 | -0.95 | -0.93 | -0.90 | 0.88 | 0.84 |
| Paranodal length | 0.93 | -0.92 | -0.95 | -0.96 | -0.95 | -0.91 | 0.93 | 0.91 |
| Paranodal gap | 0.20 | -0.16 | -0.16 | -0.21 | -0.16 | -0.11 | 0.11 | 0.22 |
| CV-CTX | 0.90 | -0.90 | -0.91 | -0.92 | -0.88 | -0.89 | 0.89 | 0.84 |
| CV-CA1 | 0.94 | -0.86 | -0.89 | -0.94 | -0.92 | -0.85 | 0.87 | 0.82 |
| CV-CA3 | 0.02 | 0.04 | 0.01 | -0.01 | -0.05 | 0.00 | 0.08 | 0.18 |
| NeuN-CTX | 0.96 | -0.95 | -0.96 | -0.97 | -0.96 | -0.93 | 0.90 | 0.88 |
| NeuN-CA1 | 0.94 | -0.90 | -0.90 | -0.93 | -0.90 | -0.90 | 0.86 | 0.86 |
| NeuN-CA3 | 0.38 | -0.41 | -0.39 | -0.38 | -0.45 | -0.33 | 0.39 | 0.18 |
