# Supplementary material for: Genetic deletion of Krüppel-like factor 11 aggravates traumatic brain injury
Source: J Neuroinflammation. 2022 Nov 19;19:281. doi: 10.1186/s12974-022-02638-0 (PMC9675068; doi:10.1186/s12974-022-02638-0)
Supplement: Supplementary file 5 — Additional file 5: Table S4. Pearson correlation analysis (p value). [file 12974_2022_2638_MOESM5_ESM.docx]

**Table S4. Pearson correlation analysis (p value)**

|  | Latency to fall | Time to touch | Time to remove | Forepaw foot fault | Hindpaw foot fault | Latency to platform | Time in target quadrant | Latency to dark box |
| --- | --- | --- | --- | --- | --- | --- | --- | --- |
| LFB-CTX | 7.44E-10 | 2.31E-10 | 5.79E-10 | 2.26E-11 | 4.47E-10 | 1.38E-08 | 8.66E-10 | 1.58E-06 |
| LFB-EC | 6.16E-11 | 4.6E-10 | 2.13E-09 | 1.58E-11 | 2.99E-09 | 9.11E-09 | 3.63E-07 | 3.2E-07 |
| LFB-STR | 7.41E-06 | 6.17E-07 | 1.21E-06 | 8.85E-07 | 3.94E-06 | 2.16E-06 | 6.68E-07 | 2.74E-05 |
| MBP-CTX | 1.6E-14 | 1.16E-10 | 1.38E-10 | 4.91E-13 | 4.94E-13 | 5.16E-10 | 1.23E-08 | 3.83E-08 |
| MBP-EC | 2.16E-11 | 9.49E-10 | 3.07E-12 | 7.91E-13 | 2.31E-12 | 4.08E-10 | 9.18E-10 | 5.35E-07 |
| MBP-STR | 4.32E-10 | 7.88E-10 | 3.08E-10 | 1.86E-10 | 7.33E-12 | 3.16E-09 | 1.93E-10 | 2.06E-07 |
| SMI32-CTX | 3.88E-11 | 1.69E-10 | 8.92E-13 | 2.2E-12 | 4.15E-11 | 2.77E-10 | 1.59E-08 | 6.78E-08 |
| SMI32-EC | 1E-11 | 3.34E-09 | 3.1E-12 | 3.89E-14 | 2.34E-15 | 4.11E-10 | 1E-09 | 8.31E-08 |
| SMI32-STR | 9.12E-13 | 3.7E-09 | 2.45E-10 | 5.9E-12 | 7.09E-11 | 2.94E-09 | 6.74E-08 | 1.29E-07 |
| SMI32/MBP-CTX | 3.91E-09 | 9.74E-08 | 1.2E-09 | 2.33E-09 | 2.03E-09 | 2.4E-09 | 1.86E-08 | 1.87E-10 |
| SMI32/MBP-EC | 1.61E-08 | 3.4E-07 | 4.66E-10 | 9.2E-10 | 7.64E-11 | 4.87E-09 | 1.46E-08 | 7.93E-08 |
| SMI32/MBP-STR | 9.65E-10 | 3.8E-08 | 3.35E-10 | 6.31E-10 | 3.77E-10 | 3.32E-11 | 2.07E-09 | 2.95E-10 |
| Number of NOR | 1.83E-14 | 6.83E-11 | 5.85E-10 | 1.02E-12 | 1.72E-11 | 8.96E-10 | 9.03E-09 | 2.06E-07 |
| Paranodal length | 1.53E-11 | 1.53E-10 | 8.3E-13 | 5.72E-14 | 3.78E-13 | 4.38E-10 | 1.73E-11 | 4.81E-10 |
| Paranodal gap | 0.337315 | 0.449748 | 0.440841 | 0.324278 | 0.442169 | 0.59878 | 0.605805 | 0.287622 |
| CV-CTX | 1.51E-09 | 2.05E-09 | 4.6E-10 | 5.72E-11 | 1.22E-08 | 5.38E-09 | 4.42E-09 | 1.69E-07 |
| CV-CA1 | 6.92E-12 | 4.26E-08 | 4.23E-09 | 4.29E-12 | 5.78E-11 | 7.8E-08 | 1.61E-08 | 7.8E-07 |
| CV-CA3 | 0.915531 | 0.828802 | 0.959655 | 0.938641 | 0.812746 | 0.994517 | 0.688665 | 0.396411 |
| NeuN-CTX | 4.6E-14 | 1.1E-12 | 2.49E-14 | 1E-16 | 6.07E-14 | 3.08E-11 | 8.93E-10 | 1.44E-08 |
| NeuN-CA1 | 2.15E-12 | 9.99E-10 | 1.29E-09 | 4.8E-11 | 1.71E-09 | 1.4E-09 | 5.56E-08 | 4.8E-08 |
| NeuN-CA3 | 0.065608 | 0.043335 | 0.059527 | 0.065419 | 0.02702 | 0.111256 | 0.052939 | 0.377419 |
